# Effects of Aging and Task Prioritization on Split-Belt Gait Adaptation

**DOI:** 10.3389/fnagi.2019.00010

**Published:** 2019-01-29

**Authors:** Danique Vervoort, A. Rob den Otter, Tom J. W. Buurke, Nicolas Vuillerme, Tibor Hortobágyi, Claudine J. C. Lamoth

**Affiliations:** ^1^Center for Human Movement Sciences, University of Groningen, University Medical Center Groningen, Groningen, Netherlands; ^2^AGEIS, University Grenoble-Alpes, Grenoble, France; ^3^Institut Universitaire de France, Paris, France

**Keywords:** split-belt walking, adaptive gait, aging, older adults, dual-task, task prioritization

## Abstract

**Background:** Age-related changes in the sensorimotor system and cognition affect gait adaptation, especially when locomotion is combined with a cognitive task. Performing a dual-task can shift the focus of attention and thus require task prioritization, especially in older adults. To gain a better understanding of the age-related changes in the sensorimotor system, we examined how age and dual-tasking affect adaptive gait and task prioritization while walking on a split-belt treadmill.

**Methods:** Young (21.5 ± 1.0 years, *n* = 10) and older adults (67.8 ± 5.8 years, *n* = 12) walked on a split-belt treadmill with a 2:1 belt speed ratio, with and without a cognitive Auditory Stroop task. Symmetry in step length, limb excursion, and double support time, and strategy variables swing time and swing speed were compared between the tied-belt baseline (BL), early (EA) and late split-belt adaptation (LA), and early tied-belt post-adaptation (EP).

**Results:** Both age groups adapted to split-belt walking by re-establishing symmetry in step length and double support time. However, young and older adults differed on adaptation strategy. Older vs. young adults increased swing speed of the fast leg more during EA and LA (0.10–0.13 m/s), while young vs. older adults increased swing time of the fast leg more (2%). Dual-tasking affected limb excursion symmetry during EP. Cognitive task performance was 5–6% lower during EA compared to BL and LA in both age groups. Older vs. young adults had a lower cognitive task performance (max. 11% during EA).

**Conclusion:** Healthy older adults retain the ability to adapt to split-belt perturbations, but interestingly age affects adaptation strategy during split-belt walking. This age-related change in adaptation strategy possibly reflects a need to increase gait stability to prevent falling. The decline in cognitive task performance during early adaptation suggests task prioritization, especially in older adults. Thus, a challenging motor task, like split-belt adaptation, requires prioritization between the motor and cognitive task to prevent adverse outcomes. This suggests that task prioritization and adaptation strategy should be a focus in fall prevention interventions.

## Introduction

Humans adapt their gait to environmental challenges, which allows walking on uneven surfaces, avoiding obstacles, and maintaining balance when slipping or tripping. Advancing age modifies the locomotor system, which reduces the ability of older adults to adapt to environmental perturbations while walking (Bierbaum et al., 2011; McCrum et al., 2016). The age-related changes include the distal-to-proximal shift in joint torques and powers (DeVita and Hortobagyi, 1985), and an increase of co-activation of agonist and antagonist lower extremity muscles (Schmitz et al., 2009). While the age-related changes increase joint stability, these changes may have negative effects, as the metabolic cost of walking increases ~20% with aging (Hortobágyi et al., 2011). Not only are there quantitative adaptations, but age also modifies the strategies used to negotiate obstacle perturbations while walking. Older adults increase step length to avoid the obstacle, which increases the sense of stability by decreasing the forward momentum of the center of mass (Weerdesteyn et al., 2005a,b).

Much less is known however about whether and how older adults adapt their gait to asymmetrical gait perturbations that are ubiquitous in daily life. A good ability to react to such perturbations could be essential to maintain walking balance and prevent falls. Perturbation studies have shown that an increase in the ability to perform reactive responses is associated with a lower number of falls (McCrum et al., 2017). A split-belt treadmill allows us to study not only the reactive responses, but also locomotor adaptations to a sustained perturbation during walking in a controlled environment (Reisman et al., 2005). By setting each belt to a different speed, both the immediate changes in step characteristics, i.e., early adaptation, and the time course of adaptation over several minutes until late adaptation can be determined.

Split-belt walking initially creates an asymmetry in step length and double support time. Young adults adapt their gait to re-establish symmetry in both step length and double support time (Reisman et al., 2005). During the entire adaptation phase there is an asymmetry in limb excursion, i.e., stride length on the split-belt treadmill (Hoogkamer et al., 2014), due to the longer stride on the fast belt during split-belt walking (Reisman et al., 2005). When returning to tied-belt walking, the so-called early post-adaptation, participants show aftereffects, such as asymmetry in step length in the opposite direction from early adaptation (Reisman et al., 2005). An inability to re-establish symmetry in step length and double support time during split-belt adaptation and the absence of aftereffects are presumable markers of reduced gait adaptability (Vasudevan et al., 2011; Bruijn et al., 2012). The early detection of reduced gait adaptability can help in identifying older adults at risk for adverse reactions to perturbations and thus prevent falls.

Healthy older adults are capable of adapting to split-belt walking, but age affects adaptation strategy, especially during the early adaptation phase. Older adults are termed as “speed” adaptors, with a ±0.15 m/s greater decrease in swing speed of the slow leg. In contrast, young adults are “timing” adapters, indicated by a ±5% shorter swing time of the slow leg. To the best of our knowledge, the previously mentioned study is the only work that examined the effects of age on gait adaptation strategies (Bruijn et al., 2012).

Besides single-task gait adaptation, dual-task gait adaptation is of interest. Distraction-free gait is rare and dual-tasking is rather the norm than the exception in daily life. There is however no consensus concerning the effects of motor-cognitive dual-tasking on gait adaptation. Dual-tasking slowed the rate of adaptation on step length symmetry in the adaptation phase in young (Malone and Bastian, 2010) and in middle-aged adults (Malone and Bastian, 2016). Motor-cognitive dual-tasking also increased stance time on the fast belt and double support time on the slow belt in young adults (McFadyen et al., 2009), and another study reported that older adults exhibited a larger step time asymmetry (Saito et al., 2013).

The inconsistent results concerning motor-cognitive dual-tasking might be due to the types of cognitive tasks performed or the gait outcomes used, but could also be due to task prioritization. An integrated task prioritization model suggests that healthy adults perform and focus on the secondary cognitive task as long as the threat to postural control is low. A challenging environment or a demanding motor task can shift the focus of attention from the secondary cognitive task to the motor task in order to maintain gait (Yogev-Seligmann et al., 2012).

While young adults can flexibly allocate attentional resources between two concurrent tasks (Raffegeau et al., 2018), attentional capacity decreases with age, thus motor-cognitive dual-tasking becomes more challenging (Huxhold et al., 2006). During dual-task split-belt walking, especially older adults may prioritize gait in order to adequately adapt to the perturbation of the split-belt. Indeed, after short perturbations while performing a cognitive task, older adults prioritized dynamic stability, as shown by a sharp increase in the number of errors on the cognitive task (Mersmann et al., 2013). While walking on elevated or narrow surfaces with a dual-task, older adults not only increased the number of errors on the cognitive task, but also committed more missteps, suggesting that prioritization of the motor task in high-risk settings might fail for older adults (Schaefer et al., 2015).

The ability to adapt to the perturbation induced by the split-belt treadmill is essential to continue walking. However, this ability might be altered or affected by age, dual-tasking or task prioritization. Therefore, our aims were to determine: (1) The effect of split-belt adaptation and age on gait strategy and symmetry; (2) The effect of motor-cognitive dual-tasking on gait adaptation, and (3) Age-related differences in task prioritization during split-belt adaptation.

We hypothesized that adaptations to split-belt walking occur independent of age, but young vs. older adults are “timing” and “speed” adaptors, respectively. We also expected that motor-cognitive dual-tasking would affect the ability to adapt to split-belt walking in the spatial or temporal gait outcomes. Furthermore, we hypothesized older vs. young adults will prioritize the motor adaptation task over the cognitive task in the most challenging early period of adaptation to split-belt walking.

## Materials and Methods

### Participants

Healthy young (21.5 ± 1.0 years, 40% male, *n* = 10) and older adults (67.8 ± 5.8 years, 58% male, *n* = 12) who could walk without aids and follow verbal instructions were included in the study. Criteria for exclusion were orthopedic, neurological, and/or psychiatric disorders that might affect gait, and prior experience with split-belt walking. The Ethical Committee of the Center of Human Movement Sciences at the University Medical Center Groningen approved the study. All participants signed a written informed consent before the measurements.

### Instrumentation and Procedure

Participants walked on an M-Gait treadmill (Motekforce Link, Amsterdam, The Netherlands), with two belts that can be controlled separately. With the embedded force plates in the treadmill, ground reaction forces were sampled at 1,000 Hz. Infrared emitting diodes were placed on the feet (5th metatarsal head) and ankle (lateral malleolus) and recorded at 100 Hz (Optotrak, Northern Digital, Ontario, Canada).

**Figure 1:**
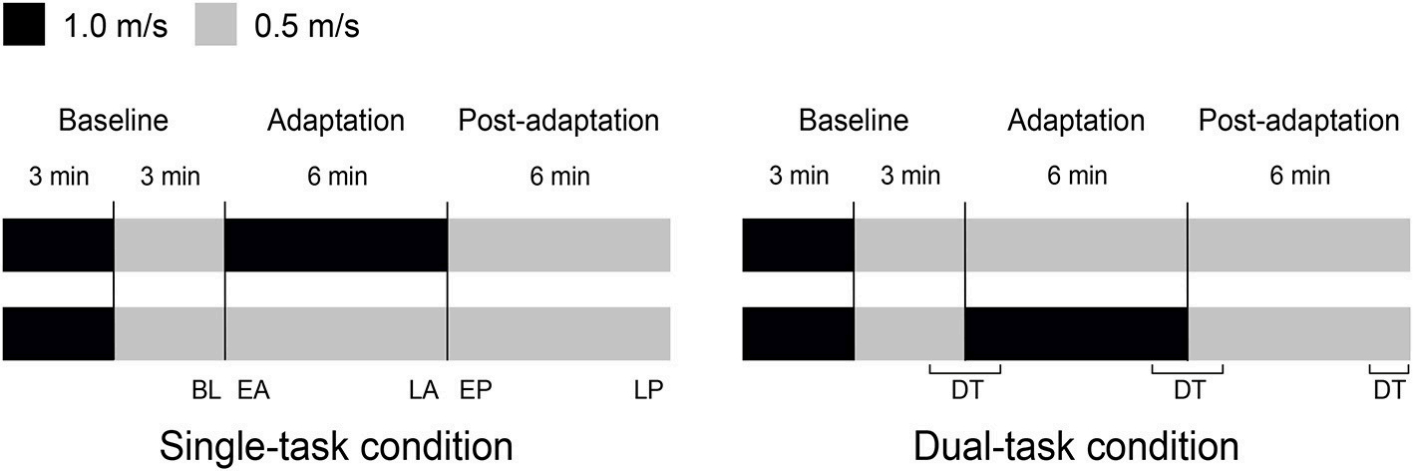
Split-belt paradigm. Healthy young and older adults walked on a split-belt treadmill programmed to speed up one of the belts from 0.5 to 1.0 m/s under single and dual-task conditions. The single-task condition was always performed before the dual-task condition. Note that the fast and slow belts were randomly assigned per participant to left or right leg for single-task walking. If the fast belt was assigned to the left belt in the single-task, then in the dual-task condition the right belt was the fast belt. The dual-task was performed during five phases: the late slow baseline (BL), early adaptation (EA), late adaptation (LA), early post-adaptation (EP), and the late post-adaptation (LP). DT, dual-task.

Participants walked on the split-belt treadmill, starting with 3 min of tied-belt walking at both 1.0 m/s and 0.5 m/s (baseline), then split-belt walking with one belt moving at 1.0 m/s and the other belt at 0.5 m/s for 6 min (adaptation) and finally with tied-belts at 0.5 m/s for 6 min (post-adaptation; Figure 1).

In the first condition (single-task), participants walked on the split-belt treadmill for a total of 18 min without a cognitive task. In the second condition (dual-task), participants walked while performing a cognitive task. This task order was chosen in order to minimize the learning effect of re-exposure to split-belt walking, which could potentially affect single-task adaptation and task prioritization during the second condition. Even though we cannot exclude that there were effects of motor learning in the second condition, due to the first condition, earlier studies have revealed that participants still show adaptation during re-exposure to the split-belt (Malone et al., 2011; Malone and Bastian, 2016), allowing us to test our hypothesis on task prioritization.

Between the two conditions, participants could rest for 2 min or longer if needed. The fast belt was randomly assigned to the left or right leg. In the dual-task condition, the fast belt was assigned to the other side as in the single-task condition, in order to minimize the learning effect of re-exposure to the split-belt.

The cognitive task was the Auditory Stroop test. The Auditory Stroop test consists of the words “high” and “low” in a high or low pitch. Participants were instructed to call out the pitch of the word they heard, ignoring the actual word presented (McFadyen et al., 2009). Participants had to be able to hear the words of the Auditory Stroop test. The test was performed in a control condition while sitting and during the dual-task condition at the last minute of the slow baseline (BL) until the first minute of adaptation (EA); the last minute of adaptation (LA) until the first minute of post-adaptation (EP); and the last minute of post-adaptation (LP; Figure 1). No instructions on task prioritization were given. Verbal responses were recorded to determine the number of correct responses.

### Data Analysis

Data were analyzed off-line with custom made Matlab codes (R2015b, The MathWorks Inc.). Vertical ground reaction forces were filtered with a second-order low-pass Butterworth filter (15 Hz cut-off). Data from the Optotrak markers were filtered with a second-order high-pass Butterworth filter (0.5 Hz cut-off).

Heel-strike and toe-off were determined at the moment the vertical forces crossed the threshold of 50 N. The foot contact moments were then used to calculate the outcome variables for the first and last five strides of the BL, EA, LA, and EP phases, allowing the following three comparisons: EA vs. BL (effect of the perturbation), LA vs. EA (adaptive change), EP vs. BL (aftereffects). For both spatial variables, limb excursion and step length, the foot contact moments were resampled to match the Optotrak sample frequency.

Symmetry variables were calculated for spatial variables step length (SL) and limb excursion (LE) and the temporal variable double support time (DS). SL was calculated as the anterior posterior distance between the ankle markers of both legs at heel-strike of the leading leg (Reisman et al., 2005). With *x* as the position of the lateral malleolus marker (latmal) at the *i*^*th*^ sample.

$$\begin{array}{r} SL_{fast}\left(i\right)=x_{latmal_{fast}}\left[t_{heelstrike\;fast}\left(i\right)\right]-x_{latmal_{slow}}\left[t_{heelstrike\;fast}\left(i\right)\right] \end{array}$$

LE was defined as the distance traveled by the ankle marker in the anterior-posterior direction from heel-strike to toe-off of one limb (Hoogkamer et al., 2014).

$$\begin{array}{r} LE\left(i\right)=x_{latmal}\left[t_{heelstrike}\left(i\right)\right]-x_{latmal}\left[t_{toeoff}\left(i\right)\right] \end{array}$$

DS was defined as the time (t) both feet were in contact with the ground (Reisman et al., 2005).

$$\begin{array}{r} DS_{fast}\left(i\right)={\text{t}}_{\text{toeoff slow}}\left(i\right)-{\text{t}}_{\text{heelstrike fast}}\left(i\right) \end{array}$$

Symmetry in SL (SLS), LE (LES), and DS (DSS) was calculated as follows (Malone and Bastian, 2010):

$$\begin{array}{r} Symmetry\;\left(i\right)=\frac{Fast\left(i\right)-Slow\left(i\right)\;}{Fast\left(i\right)+Slow\left(i\right)} \end{array}$$

To reduce the SL asymmetry induced by the split-belt perturbation, the fast leg needs to be placed further forward than the slow leg. This can be achieved by using two strategies: 1) increasing the time spent in swing, or 2) increasing swing speed. Therefore, the strategy variables percentage swing time (SwT) and swing speed (SwS) were calculated as (Bruijn et al., 2012):

$$\begin{array}{rcl} SwT_{fast/slow}\left(i\right) & = & \frac{t_{heelstrike\left(i\right)}-t_{toeoff\left(i\right)}}{t_{heelstrike\left(i+1\right)}-t_{heelstrike\left(i\right)}}\times 100\\ SwS_{fast/slow}\left(i\right) & = & \frac{LE\left(i\right)}{t_{heelstrike\left(i\right)}-t_{toeoff\left(i\right)}} \end{array}$$

For all gait variables, the dual-task cost (DTC) was determined from the single and dual-task condition values, with DTC values above zero indicating a larger value in the single-task condition and values below zero indicating that there was an effect of the dual-task (Raffegeau et al., 2018).

$$\begin{array}{r} DTC=\frac{\left(single\;task-dual\;task\right)}{single\;task}*100 \end{array}$$

Cognitive task performance (CTP) was calculated with the following formula, with n as the number of stimuli. A values of one indicates a perfect performance.

$$\begin{array}{r} CTP=\frac{n_{correct}}{n_{total}} \end{array}$$

### Statistical Analysis

To examine the effect of split-belt walking and if young and older adults differ on split-belt adaptation (aim 1), a repeated measures ANOVA was conducted with within factor Phase (BL-EA-LA-EP) and between factor Group (young vs. older adults) during single-task split-belt walking for the dependent gait variables. When a main effect of phase was found, a *post-hoc* dependent samples *t*-test with Holm-Bonferroni correction was applied to the following phase comparisons: EA vs. BL (effect of the perturbation), LA vs. EA (adaptive change), EP vs. BL (aftereffects). For significant interaction effects, the difference between age groups was tested with an independent samples *t*-test for each phase separately.

To address the second aim, the differences between single and dual-task split-belt walking were assessed using planned comparison *t*-tests to determine if DTC was different from zero for each of the four phases (BL-EA-LA-EP).

For the third aim, the differences in prioritization during dual-task split-belt adaptation between young and older adults, adaptation effects were first tested with a repeated measures ANOVA for the dual-task condition with within factor Phase (BL-EA-LA-EP) and between factor Group (young vs. older adults) for all gait variables. *Post-hoc* testing was done similarly as for the first aim. The cognitive task performance was tested with a similar repeated measures ANOVA with the CTP as dependent variable. Differences between the age groups during the control task (sitting) were tested with an independent samples *t*-test to test if there were differences between the age groups during the single task. Statistical analysis was performed using SPSS (24.0, IBM Corp. Armonk, NY, USA). Level of significance was set at *p* < 0.05.

## Results

### Single-Task Split-Belt Adaptation and Differences Between Young and Older Adults

**Figure 2:**
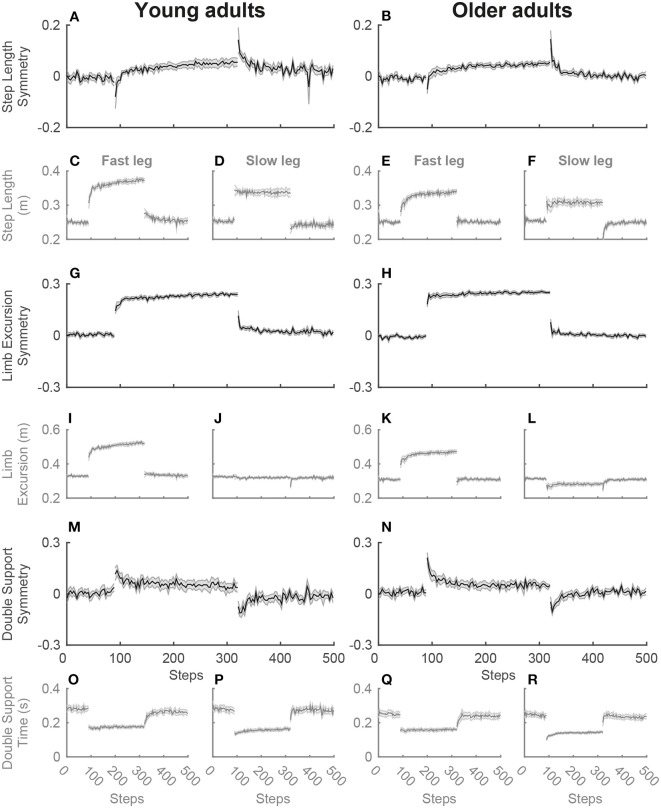
Adaptation plots of the symmetry variables for young and older adults during the single-task split-belt condition. The adaptation plot shows the development of the mean and standard deviation of the symmetry variables over the split-belt condition, starting with the 90 steps of the slow baseline, then 230 steps of the adaptation phase and then the post-adaptation phase with 180 steps. The symmetry values for step length **(A,B)**, limb excursion **(G,H)**, and double support time **(M,N)** and the separate values for the fast and slow leg [step length **(C–F)**, limb excursion **(I–L)**, and double support time **(O–R)**] are shown for young (left) and older adults (right).

**Figure 3:**
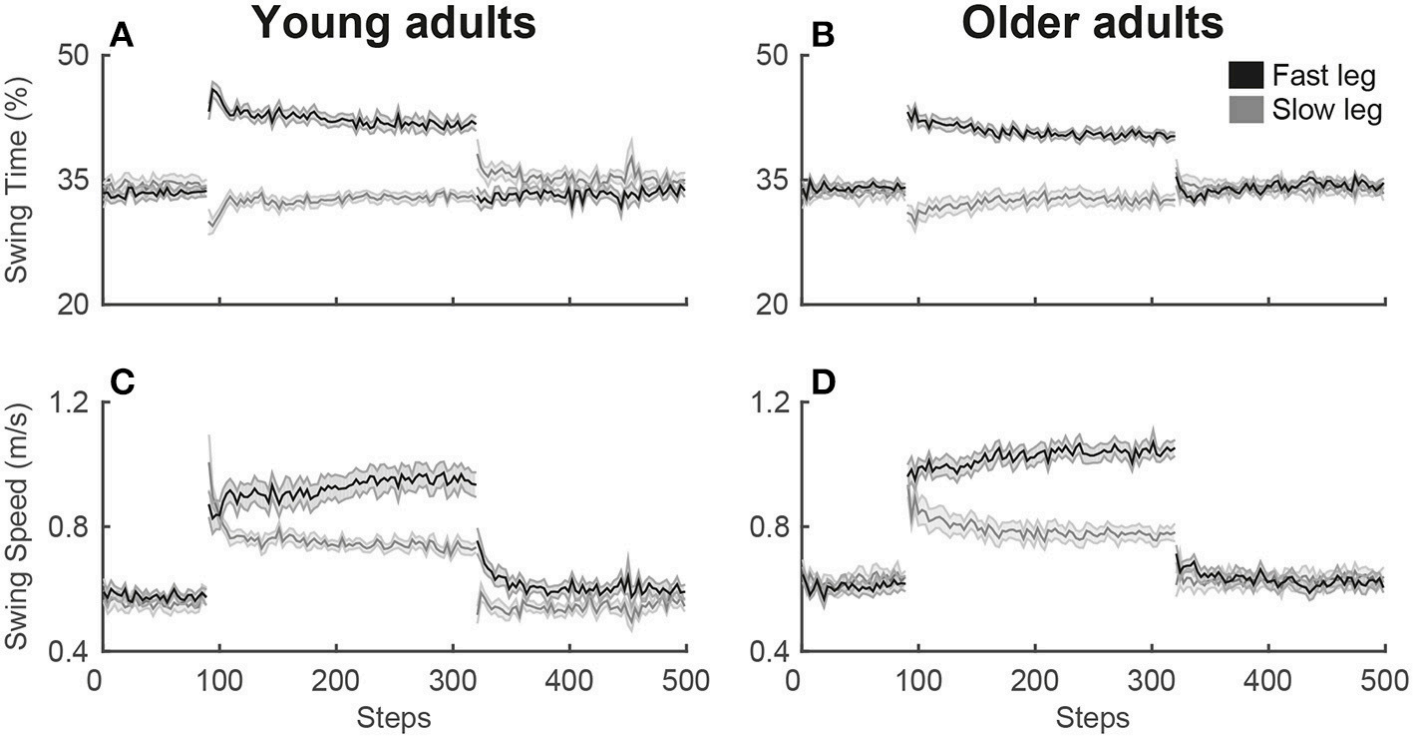
Adaptation plots of the strategy variables for young and older adults during the single-task split-belt condition. The adaptation plot shows the development of the mean and standard deviation of the strategy variables swing time **(A,B)** and swing speed **(C,D)** over the split-belt condition. The adaptation plot starts with the 90 steps of the slow baseline, then 230 steps of the adaptation phase and then the post-adaptation phase with 180 steps. Values of the fast leg are shown in black, values of the slow leg are shown in gray.

For the single-task split-belt condition, a significant main effect of phase was found for the symmetry variables, step length symmetry [SLS; *F*_(2.3, 47)_ = 28.3; *p* < 0.001], limb excursion symmetry [LES; *F*_(1.8, 35)_ = 130.8; *p* < 0.001] and double support symmetry [DSS; *F*_(2.1, 42)_ = 46.0; *p* < 0.001], as well as for the strategy variables, swing time of the fast [SwT_fast_; *F*_(3, 60)_ = 122.6; *p* < 0.001] and slow leg [SwT_slow_; *F*_(2.3, 46)_ = 23.0; *p* < 0.001] and swing speed of the fast [SwS_fast_; *F*_(3, 60)_ = 102.5; *p* < 0.001] and slow leg [SwS_slow_; *F*_(2.2, 44)_ = 64.1; *p* < 0.001]. *Post-hoc* testing revealed that for the symmetry variables SLS and DSS, an asymmetry occurred in early adaptation (EA) due to the perturbation of the changing belt speeds, while symmetry was re-established in late adaptation (LA). The early post-adaptation phase (EP) showed aftereffects of SLS and DSS asymmetry in the opposite direction from early adaptation. During the entire adaptation phase, both early and late adaptation, there is an asymmetry in LES due to the longer stride on the fast belt (see Figure 2). For the strategy variables, an increase was seen in swing time of the fast leg and swing speed for both legs during early adaptation, while there was a decrease in swing time of the slow leg. While swing time of the fast leg and swing speed of the slow leg slightly decreased until late adaptation, swing speed of the fast leg continued to increase (see Figure 3). Table 1 presents the direction of the changes of the gait variables over the phases and the corresponding *post-hoc* statistics.

**Table 1:** *Post-hoc* tests for the main effect of phase in the single-task split-belt condition.

***Post*****-*****hoc*** **tests for the main effect of phase**									
**Variable**	**EA - BL**			**LA - EA**			**EP - BL**		
	**Diff**	***t***_**(21)**_	***p***	**Diff**	***t***_**(21)**_	***p***	**Diff**	***t***_**(21)**_	***p***
SLS	−0.04, 0.09	−1.90	0.07	0.10, 0.07	6.04	**<0.001**	0.13, 0.09	6.72	**<0.001**
LES	0.19, 0.07	13.45	**<0.001**	0.06, 0.05	5.62	**<0.001**	0.08, 0.06	6.02	**<0.001**
DSS	0.15, 0.10	6.73	**<0.001**	−0.12, 0.11	−5.27	**<0.001**	−0.09, 0.08	−5.43	**<0.001**
SwT_fast_ (%)	9.34, 3.59	12.19	**<0.001**	−2.14, 2.46	−4.09	**0.001**	−0.09, 2.58	−0.16	0.87
SwT_slow_ (%)	−3.53, 3.33	−4.96	**<0.001**	2.30, 2.26	4.76	**<0.001**	2.21, 3.94	2.62	**0.016**
SwS_fast_ (m/s)	0.32, 0.14	11.19	**<0.001**	0.06, 0.12	2.45	**0.023**	0.12, 0.10	5.39	**<0.001**
SwS_slow_ (m/s)	0.32, 0.18	8.12	**<0.001**	−0.17, 0.14	−5.50	**<0.001**	−0.01, 0.10	−0.68	0.51

*The table presents results of the post-hoc t-tests, with the t-value [t_(df)_], the p-value and the differences (Diff) between the two phases that were tested. Diff values are mean ± standard deviations. P-values are highlighted in bold if the phases were significantly different after the Holm-Bonferroni correction. Diff, difference score; BL, baseline; EA, early adaptation; LA, late adaptation, EP, early post-adaptation; SLS, step length symmetry; LES, limb excursion symmetry; DSS, double support symmetry; SwT, swing time; SwS, swing speed*.

**Figure 4:**
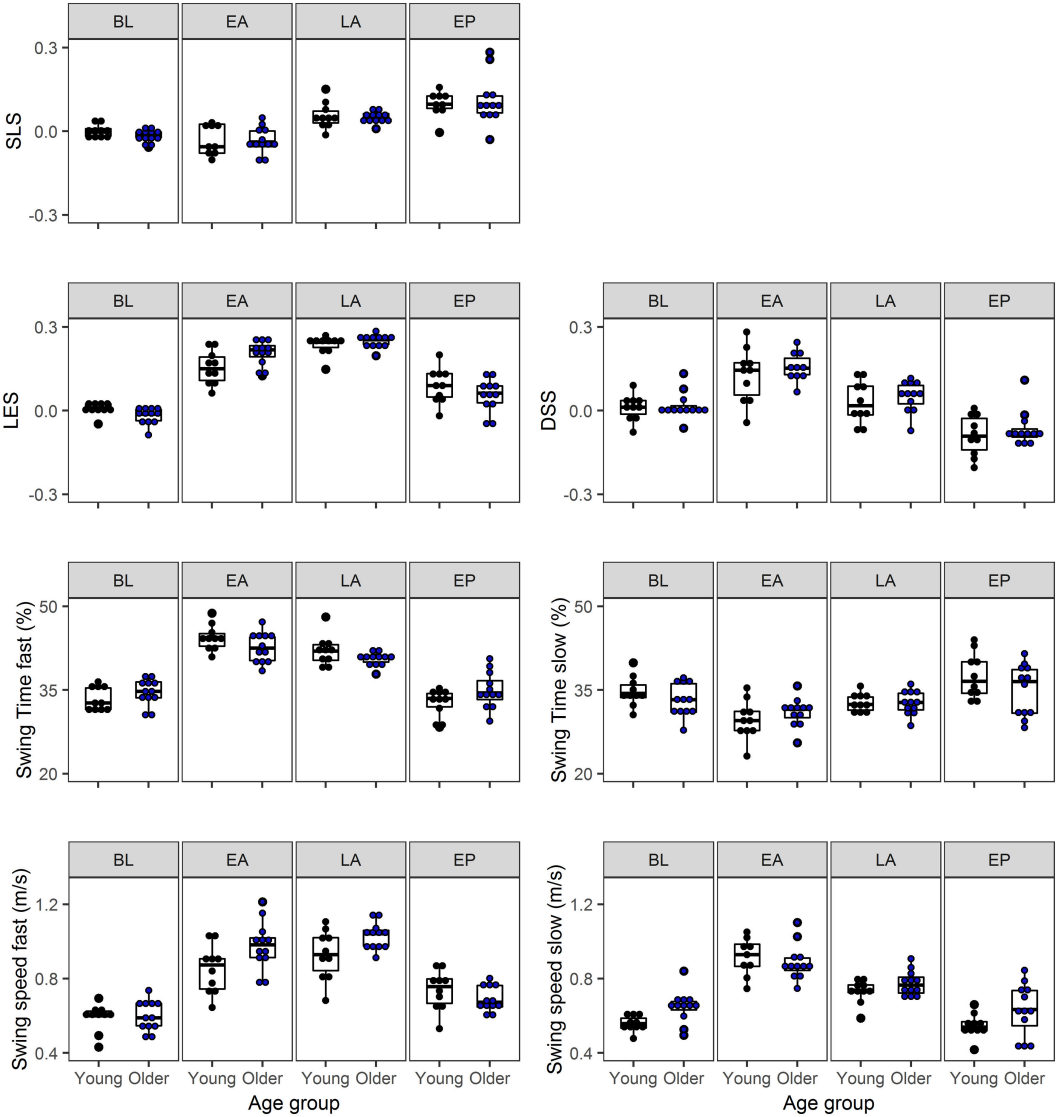
Bar plots of the symmetry (SLS, LES, DSS) and the strategy variables (Swing time, Swing speed) for young and older adults on the single-task split-belt condition. The four phases: baseline (BL), early adaptation (EA), late adaptation (LA), and early post-adaptation (EP) are shown next to each other. The bar plots show the median, the upper, and lower quartiles and the min and max value of the age groups. The dots show the individual data of the young (black) and older adults (blue). There were interaction effects for LES, Swing time of the fast leg, and Swing speed of both legs. SLS, step length symmetry; LES, limb excursion symmetry; DSS, double support symmetry.

There were no main effects of group for the gait variables, but Phase by Group interactions were significant for LES [*F*_(1.8, 35)_ = 4.4; *p* = 0.024], SwT_fast_ [*F*_(3, 60)_ = 4.8; *p* = 0.006], SwS_fast_ [*F*_(3, 60)_ = 5.3; *p* = 0.003], and SwS_slow_ [*F*_(2.2, 44)_ = 4.2; *p* = 0.018]. *Post-hoc* testing revealed that during baseline (BL) there was a slight asymmetry of LES in opposite directions in young and older adults [*t*_(20)_ = 2.3; *p* = 0.033; see Figure 4]. In early adaptation there was a difference of 0.06 greater asymmetry in LES in older vs. young adults [*t*_(20)_ = −2.5; *p* = 0.023]. SwT_fast_ showed that young adults had a trend of ±2% higher swing time of the fast leg compared to older adults during early [*t*_(20)_ = 1.8; *p* = 0.094] and late adaptation [*t*_(20)_ = 1.7; *p* = 0.100]. During both early and late adaptation older vs. young adults had, respectively a 0.13 and 0.10 m/s higher swing speed of the fast leg [respectively *t*_(20)_ = −2.3; *p* = 0.034 and *t*_(20)_ = −2.3; *p* = 0.032; see Figure 4]. Older compared to young adults had a 0.09 m/s higher swing speed of the slow leg during baseline [*t*_(20)_ = −3.0; *p* = 0.006].

### Difference Between Single and Dual-Task Split-Belt Walking

No significant effects for dual task cost were found between the single and dual-task condition, except for LES during early post-adaptation [*t*_(21)_ = 2.3; *p* = 0.033]. During motor-cognitive dual-tasking, the aftereffects of LES were lower than during the single-task.

### Dual-Task Split-Belt Adaptation for Young and Older Adults

**Figure 5:**
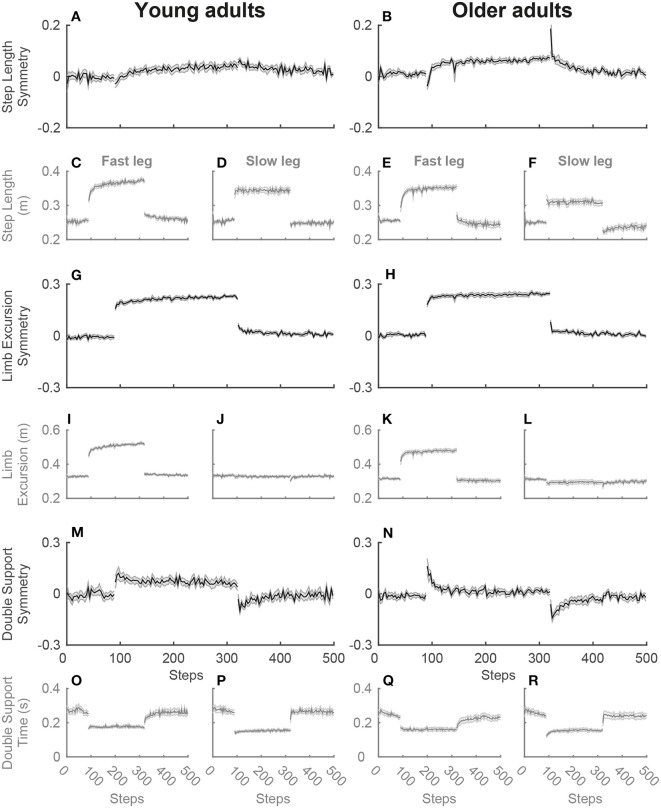
Adaptation plots of the symmetry variables for young and older adults during the dual-task split-belt condition. The adaptation plot shows the development of the mean and standard deviation of the symmetry variables over the split-belt condition, starting with the 90 steps of the slow baseline, then 230 steps of the adaptation phase and then the post-adaptation phase with 180 steps. The symmetry values for step length **(A,B)**, limb excursion **(G,H)**, and double support time **(M,N)** and the separate values for the fast and slow leg [step length **(C–F)**, limb excursion **(I–L)**, and double support time **(O–R)**] are shown for young (left) and older adults (right).

**Figure 6:**
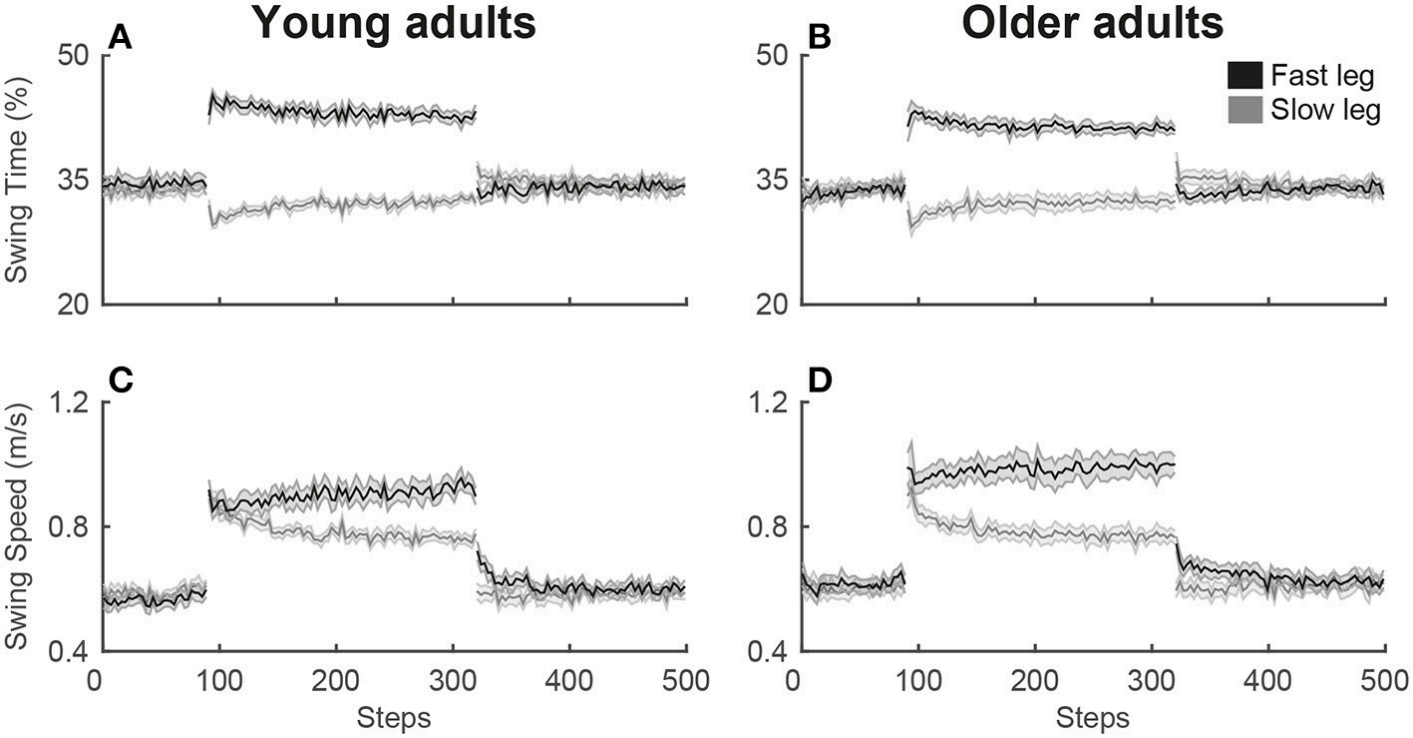
Adaptation plots of the strategy variables for young and older adults during the dual-task split-belt condition. The adaptation plot shows the development of the mean and standard deviation of the strategy variables swing time **(A,B)** and swing speed **(C,D)** over the split-belt condition. The adaptation plot starts with the 90 steps of the slow baseline, then 230 steps of the adaptation phase and then the post-adaptation phase with 180 steps. Values of the fast leg are shown in black, values of the slow leg are shown in gray.

There was a significant main effect of Phase for both symmetry, SLS [*F*_(2.3, 47)_ = 28.3; *p* < 0.001], LES [*F*_(1.8, 35)_ = 130.8; *p* < 0.001] and DSS [*F*_(2.1, 42)_ = 46.0; *p* < 0.001], and strategy gait variables, SwT_fast_ [*F*_(3, 60)_ = 122.6; *p* < 0.001], SwT_slow_ [*F*_(2.3, 46)_ = 23.0; *p* < 0.001], SwS_fast_ [*F*_(3, 60)_ = 102.5; *p* < 0.001], and SwS_slow_ [*F*_(2.2, 44)_ = 64.1; *p* < 0.001], similar to the single-task condition. *Post-hoc* testing revealed that the symmetry variables SLS and DSS showed the similar pattern of asymmetry during early adaptation and re-established symmetry during late adaptation, with opposite aftereffects during early post-adaptation (see Table 2 for the direction and *post-hoc* statistics of all gait variables). Thus, there was still adaptation during this second exposure of split-belt walking necessary for testing task prioritization (see Figures 5, 6). No significant age or interaction effects were found in any of the gait variables.

**Table 2:** *Post-hoc* tests for the main effect of phase in the dual-task split-belt condition.

***Post*****-*****hoc*** **tests for the main effect of phase**									
**Variable**	**EA - BL**			**LA - EA**			**EP - BL**		
	**Diff**	***t***_**(21)**_	***p***	**Diff**	***t***_**(21)**_	***p***	**Diff**	***t***_**(21)**_	***P***
SLS	−0.03, 0.05	−2.45	**0.023**	0.10, 0.04	11.28	**<0.001**	0.11, 0.14	3.57	**0.002**
LES	0.17, 0.04	19.31	**<0.001**	0.06, 0.03	8.72	**<0.001**	0.05, 0.03	7.96	**<0.001**
DSS	0.12, 0.13	4.04	**0.001**	−0.11, 0.13	−3.83	**0.001**	−0.08, 0.10	−3.75	**0.001**
SwT_fast_ (%)	9.01, 3.77	11.21	**<0.001**	−1.77, 3.87	−2.15	0.04	−0.05, 1.95	−0.13	0.90
SwT_slow_ (%)	−3.40, 2.80	−5.70	**<0.001**	1.99, 3.54	2.64	**0.015**	1.98, 2.38	3.91	**0.001**
SwS_fast_ (m/s)	0.32, 0.14	11.14	**<0.001**	0.06, 0.15	1.80	0.09	0.09, 0.07	5.61	**<0.001**
SwS_slow_ (m/s)	0.29, 0.08	16.84	**<0.001**	−0.14, 0.10	−6.61	**<0.001**	−0.02, 0.09	−0.80	0.43

*The table presents results of the post-hoc t-test, with the t-value [t_(df)_], the p-value and the differences (Diff) between the two phases that were tested. Diff values are mean ± standard deviations. P-values are highlighted in bold if the phases were significantly different after the Holm-Bonferroni correction. Abbreviations: Diff, difference score; BL, baseline; EA, early adaptation; LA, late adaptation; EP, early post-adaptation; SLS, step length symmetry; LES, limb excursion symmetry; DSS, double support symmetry; SwT, swing time; SwS, swing speed*.

### Differences in Cognitive Task Performance Between Young and Older Adults

**Figure 7:**
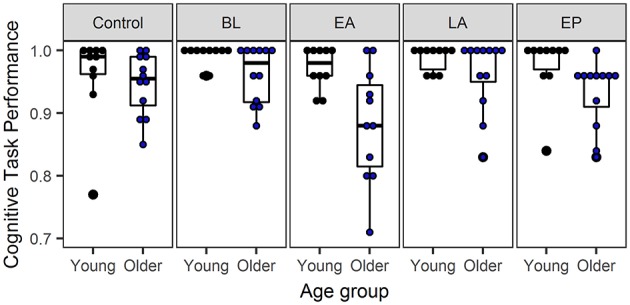
Bar plot of the cognitive task performance for young and older adults. Values are shown for the control condition, sitting, and the four phases of the split-belt paradigm: baseline (BL), early adaptation (EA), late adaptation (LA), and early post-adaptation (EP). The bar plots show the median, the upper, and lower quartiles and the min and max value of the age groups. The dots show the individual data of the young (black) and older adults (blue). A value of one on cognitive task performance indicates a perfect performance.

In the seated control condition, the two age groups did not differ in cognitive task performance (CTP; Young: 0.95 ± 0.05, Old: 0.96 ± 0.07; see Figure 7).

During motor-cognitive dual-tasking, there was a significant main effect of phase for CTP [*F*_(2.3, 46)_ = 6.5; *p* = 0.002]. *Post-hoc* testing showed that all adults made on average 5–6% more errors on the Auditory Stroop task during early adaptation compared to their performance during baseline [*t*_(21)_ = 3.0; *p* = 0.006] and late adaptation [*t*_(21)_ = −3.1; *p* = 0.006]. There was a significant main effect of group on CTP over all the split-belt phases (BL-EA-LA-EP). Young vs. old adults performed better on the cognitive task [*F*_(1, 20)_ = 11.0; *p* = 0.003; see Figure 7], with the largest difference of 11% seen in EA.

## Discussion

The overall aim of the present study was to gain insight into the effects of age and dual-tasking on adaptation to a perturbation induced by a split-belt treadmill and task prioritization. More specifically, we examined the effects of age and dual-tasking on gait symmetry and strategy.

Both young and older adults adapted to split-belt walking and re-established gait symmetry, but the two age groups achieved this using a different strategy. Older adults increased swing speed of the fast leg, whereas young adults showed a trend of increased swing time of the fast leg. Dual-tasking compared with single-task split-belt walking did not affect gait adaptation strategies, but only affected limb excursion symmetry during the early post-adaptation phase, as indicated by smaller aftereffects. The lack of dual-task effects on all other gait variables and phases is likely due to task prioritization. Task prioritization is clearly present within the dual-task condition in the early adaptation phase, as revealed by a worse cognitive task performance, even more so for older compared to young adults. We discuss these results with a perspective on how healthy older adults retain the ability to adapt to the split-belt perturbation.

In line with results of previous research (Reisman et al., 2005; Malone and Bastian, 2016), both young and older adults re-established symmetry in step length and double support time after the initial perturbation. The re-established symmetry by the older adults in this study indicate a retained ability to continuously adapt to split-belt walking. Although symmetry variables are widely reported and nicely reflect short as well as longer term split-belt adaptation, it is nevertheless important to examine the contribution of both legs (fast and slow) to clearly show the effects of gait adaptation (Hoogkamer et al., 2014). The effects on gait symmetry can be harder to distinguish at lower speeds or speed ratios, like the 1:2 speed ratio of 0.5–1 m/s used in this study for feasibility. In the current study, we found most of the well-documented adaptation effects on gait symmetry, even with the limitations of these lower speeds and a small sample size with some variation within the age groups (see Figure 4). It would therefore be interesting to further examine adaptive gait and the variation between participants with a larger sample size at slightly higher speeds to show even clearer differences within and between groups.

While both age groups adapt to split-belt walking, our results confirm previous data showing that the adaptation strategies were age-dependent (Bruijn et al., 2012). Swing time of the fast leg showed a trend with a 2% larger increase for young compared to older adults, while swing speed of the fast leg increased 0.10–0.13 m/s more for older than young adults. With the limitation of a small sample size in the current study, only a trend of adaptation in ‘timing’ for young adults was found, but a larger sample size in future studies could further confirm the adaptation strategies. The current results do agree with the concept that adaptations occur in ‘timing’ and ‘speed’ in young and older adults, respectively (Bruijn et al., 2012). However, we found the ‘timing’ and ‘speed’ effects for the fast instead of the slow leg, possibly due to the treadmill being stopped between the baseline and adaptation phase in the previous study, while our participants continued walking. This causes a difference in acceleration of the belts between the two studies in that in our study only one belt accelerated with max 0.5 m/s^2^, while in the previous study both belts accelerated with max 0.3 m/s^2^ (Bruijn et al., 2012).

The age-related change in adaptation strategy might be beneficial for older adults. Switching from a time to a speed strategy in response to a split-belt perturbation decreases the time spent in swing and thus decreases single leg stance, which increases gait stability during split-belt walking, especially immediately after the perturbation (Bruijn et al., 2012). Maintaining dynamic stability during split-belt walking is also important over the longer period of adaptation, as shown by the adaptation of gait stability during the continuous perturbation of split-belt walking (Park and Finley, 2017; Buurke et al., 2018). Furthermore, asymmetry in gait is associated with poor dynamic stability in stroke survivors (Lewek et al., 2014). In the context of the present study, the age-related differences in strategy to re-establish gait symmetry might be essential for older adults to maintain gait stability while walking on the split-belt to prevent falling.

Beyond gait stability, age-related decreases in neuromuscular function could contribute to the age-related differences in timing and speed strategies. The age-related reductions in leg muscle strength (Nigg et al., 1994; Hayashida et al., 2014) and power (Bean et al., 2002) are associated with changes in the walking pattern including a slowing of gait speed, an increase in stance and double support time, and a decrease in swing time (Winter et al., 1990; Samson et al., 2001; Laufer, 2005). The neuromuscular changes might thus limit swing time during split-belt walking in old adults. Future research should determine the relationship between the age-related changes in neuromuscular function and the gait adaptation strategies observed in the present study. A comprehensive analysis of muscle activation patterns during split-belt walking, in both timing and contributions of muscles, could provide further insights into neuromuscular mechanisms underlying the age-dependent variations in gait adaptations.

Dual-tasking did not affect gait adaptation as there were no significant effects on dual-task cost, except for the aftereffects of limb excursion symmetry. The smaller aftereffects could be due to the fact that the dual-task caused participants to retain less of the split-belt adaptation. Since there was no randomization between the two conditions, we cannot exclude that fatigue might have had effects on the dual-task results, which is a limitation of the study. The lack of further dual-task effects is in contrast to the previously discussed motor-cognitive dual-tasking studies during split-belt walking (McFadyen et al., 2009; Malone and Bastian, 2010, 2016; Saito et al., 2013). We however propose that participants prioritized gait over the cognitive task, minimizing any effects of the secondary cognitive task on the gait variables.

At the onset of the speed differences between the belts, the early adaptation phase, both young and older adults performed worse on the cognitive task, as indicated by fewer responses to the Auditory Stroop stimuli compared to baseline and late adaptation. This result suggests that immediately after being exposed to split-belt walking there is a need to prioritize gait over performing the cognitive task for the sake of safety. Our findings support the theory of task prioritization (Yogev-Seligmann et al., 2012) that people tend to prioritize the motor task over the cognitive task in a more challenging environment, in this case, a challenging motor task, which requires attentional capacity to maintain gait.

Furthermore, during dual-task split-belt walking older vs. young adults had 3–11% fewer correct responses on the cognitive task, with the largest differences also during the early adaptation phase. This implies that performing an additional task while reacting to the split-belt perturbation is harder for older adults. Therefore, it seems that older adults even more than young adults need to prioritize gait. The increased need to prioritize gait may be due to age-related changes. Interference from dual-tasking on walking ability starts at a lower level for individuals with less task-relevant resources, like older adults (Huxhold et al., 2006). Older adults might also have a higher need to focus their attention on foot placement in response to the split-belt perturbation, since afferent feedback is impaired in old age (Goble et al., 2009). Afferent feedback facilitates adaptive gait, and a reduction in feedback from muscles leads to a poorer detection of errors that are important for accurate gait adaptation (Bastian, 2011). By prioritizing the motor task over the cognitive task during split-belt walking, older adults show that they have retained the ability to compensate for the age-related neuromuscular decline, which could help in preventing adverse reactions to perturbations.

## Conclusions

Age did not affect gait symmetry after a split-belt perturbation, but did affect adaptation strategy, with young and older adults adapting through “timing” and “speed,” respectively. The role of this change in adaptation strategy is likely to increase gait stability in older adults to prevent falling. Task prioritization of the motor over the cognitive task may underlie the lack of dual-tasking effects on gait adaptation. This is supported by a decline in cognitive task performance during early adaptation, more so in older compared with young adults. We conclude that healthy older adults retain the ability to adapt to split-belt perturbations, but interestingly they adapt through a different adaptation strategy. Moreover, in challenging motor tasks, like split-belt adaptation, this requires task prioritization to prevent adverse outcomes. This suggests that task prioritization and adaptation strategy should be a focus in fall prevention interventions. Furthermore, future research should determine the relationship between gait adaptation strategies, gait stability, and neuromuscular function to understand the underlying mechanisms of age-related differences in split-belt adaptation.

## Availability of Data and Material

The data set used and analyzed in the current study is available from the corresponding author on reasonable request.

## Author Contributions

DV, CL, TH, and NV designed the study protocol. DV collected and analyzed the data with supervision of CL. Results were interpreted by DV, AdO, NV, TB, TH, and CL. DV wrote the first draft under supervision of CL. DV, CL, AdO, NV, TB, and TH contributed significantly to revising the manuscript. All authors read and approved the final manuscript.

### Conflict of Interest Statement

The authors declare that the research was conducted in the absence of any commercial or financial relationships that could be construed as a potential conflict of interest.

## Abbreviations

BL: late slow baseline
EA: early adaptation
LA: late adaptation
EP: early post-adaptation
SL: step length
LE: limb excursion
DS: double support time
SLS: step length symmetry
LES: limb excursion symmetry
DSS: double support symmetry
SwT: swing time
SwS: swing speed
DTC: dual-task cost
CTP: cognitive task performance.
